# Application effect of an artificial intelligence-based fundus screening system: evaluation in a clinical setting and population screening

**DOI:** 10.1186/s12938-023-01097-9

**Published:** 2023-04-24

**Authors:** Shujuan Cao, Rongpei Zhang, Aixin Jiang, Mayila Kuerban, Aizezi Wumaier, Jianhua Wu, Kaihua Xie, Mireayi Aizezi, Abudurexiti Tuersun, Xuanwei Liang, Rongxin Chen

**Affiliations:** 1 State Key Laboratory of Ophthalmology, Guangdong Provincial Key Laboratory of Ophthalmology and Visual Science, Guangdong Provincial Clinical Research Center for Ocular Diseases, Zhongshan Ophthalmic Center, Sun Yat-sen University, Guangzhou, 510060 China; 2 Ophthalmologic Center, The Affiliated Kashi Hospital of Sun Yat-sen University, The First People’s Hospital of Kashi Prefecture, Kashi, 844000 China

**Keywords:** Artificial intelligence, Color fundus photography, Ocular fundus abnormalities, Early screening, Prevention of blindness

## Abstract

**Background:**

To investigate the application effect of artificial intelligence (AI)-based fundus screening system in real-world clinical environment.

**Methods:**

A total of 637 color fundus images were included in the analysis of the application of the AI-based fundus screening system in the clinical environment and 20,355 images were analyzed in the population screening.

**Results:**

The AI-based fundus screening system demonstrated superior diagnostic effectiveness for diabetic retinopathy (DR), retinal vein occlusion (RVO) and pathological myopia (PM) according to gold standard referral. The sensitivity, specificity, accuracy, positive predictive value (PPV) and negative predictive value (NPV) of three fundus abnormalities were greater (all > 80%) than those for age-related macular degeneration (ARMD), referable glaucoma and other abnormalities. The percentages of different diagnostic conditions were similar in both the clinical environment and the population screening.

**Conclusions:**

In a real-world setting, our AI-based fundus screening system could detect 7 conditions, with better performance for DR, RVO and PM. Testing in the clinical environment and through population screening demonstrated the clinical utility of our AI-based fundus screening system in the early detection of ocular fundus abnormalities and the prevention of blindness.

## Background

Ocular fundus abnormalities are the essential causes of blindness and can be induced by ocular fundus diseases as well as other eye diseases such as glaucoma. Notably, diabetic retinopathy (DR), the primary cause of blindness and visual impairment among working age people, causes more than 24,000 people to lose their vision [1]. Age-related macular degeneration (ARMD), whose etiology is dependent on both hereditary and environmental factors, is the most common cause of significant vision impairment in adults who are equal to or older than 50 years old [2]. Retinal vein occlusion (RVO), the second most prevalent blinding vascular retinal disorder after DR [3], is thought to affect up to 16.4 million individuals worldwide, with a frequency of 2.1% in those over the age of 40 [4]. One of the main causes of blindness in young adults is pathological myopia (PM), which occurs in high myopic individuals with posterior scleral staphyloma and maculopathy [5]. The leading cause of ocular illnesses that result in blindness is glaucoma [6], and it is predicted that the number of glaucoma patients may reach 111.8 million by 2040 [7]. All of these findings show that aberrations of the ocular fundus can occur at any age and typically results in irreversible blindness. For ocular fundus abnormalities, early detection and identification are crucial for prevention and treatment due to the gradual destruction of ocular fundus structure and function [8].

Based on ocular imaging technologies, AI screening systems have the advantages of being simple to use, resource saving, and appropriate for use in primary regions [9]. These systems have the potential to become a future trend in ocular fundus abnormality screening, particularly in primary regions, as a result of its ability to assist primary healthcare facilities in diagnosing patients with ocular fundus abnormalities earlier and providing patients with treatment options or advice on referrals. Internationally, there is no widely used AI-based fundus screening system. The robust learning capacity of our transfer learning algorithm has been proved by retinal optical coherence tomography (OCT) images and chest radiographs [10]. Our transfer learning system could give highly effective classifications even under a very limited training dataset [10]. Notably, color fundus photography (CFP) is safe and effective and has a higher diagnostic efficiency even for glaucoma [11]. Kashi Prefecture is a significant area for the prevention of blindness and the ideal location for our AI screening system test due to its huge population base and dispersed population distribution. We used our AI screening system that can classify 7 conditions, including 5 common ocular fundus abnormalities, ARMD, DR, RVO, PM and glaucoma, to evaluate the viability of the system in this region and the efficacy of its application in primary population screening.

## Results

### Basic information

A total of 637 color fundus images from the ophthalmic clinic and 20,355 images from the physical examination center were collected for the evaluation in the clinical environment and the population screening, respectively. For the evaluation in the clinical environment, there were 158 males (48.32%) and 169 females (51.68%). Their ages ranged between 4 and 87, with an average of 48.18 ± 18.83 years old. For the population screening, there were 5481 males (52.52%) and 4956 females (47.48%). Their ages ranged between 2 and 103 years old, with the average of 46.31 ± 15.78 years old (Table 1).

**Table 1:** Characteristics of patients whose color fundus photographs were included in the study

Application environment	Normal	ARMD	DR	RVO	Referable glaucoma	PM	Other abnormalities
Ophthalmic clinic							
Number of patients	230	11	9	14	35	24	61
Mean age (years)	47.77 ± 19.63 (Range: 4–84)	67.27 ± 8.30 (Range: 51–81)	61.44 ± 5.64 (Range: 52–69)	55.93 ± 14.07 (Range: 13–71)	56.46 ± 15.13 (Range: 11–87)	38.12 ± 16.90 (Range: 9–69)	47.13 ± 16.75 (Range: 8–79)
Sex							
Male	95 (41.30%)	8 (72.73%)	6 (66.67%)	5 (35.71%)	20 (57.14%)	13 (54.17%)	35 (57.38%)
Female	135 (58.70%)	3 (27.27%)	3 (33.33%)	9 (64.29%)	15 (42.86%)	11 (45.83%)	26 (42.62%)
Ethnicity							
Han	18 (7.83%)	1 (9.09%)	1 (11.11%)	0	1 (2.86%)	2 (8.33%)	2 (3.28%)
Uyghur	209 (90.87%)	10 (90.91%)	8 (88.89%)	14 (100%)	34 (97.14%)	22 (91.67%)	59 (96.72%)
Other	3 (1.30%)	0	0	0	0	0	0
Physical examination center							
Number of patients	8349	509	68	254	1548	334	199
Mean age (years)	46.20 ± 15.46 (Range: 3–103)	51.58 ± 16.16 (Range: 40–99)	56.9 ± 14.47 (Range: 20–82)	55.23 ± 12.56 (Range: 11–82)	49.91 ± 16.89 (Range: 3–92)	48.43 ± 16.77 (Range: 10–84)	51.07 ± 17.13 (Range: 4–87)
Sex							
Male	4340 (51.98%)	279 (54.81%)	30 (44.12%)	137 (53.94%)	894 (57.75%)	153 (45.81%)	116 (58.29%)
Female	4009 (48.02%)	230 (45.19%)	38 (55.88%)	117 (46.06%)	654 (45.25%)	181 (54.19%)	83 (41.71%)
Ethnicity							
Han	661 (7.92%)	38 (7.47%)	6 (8.82%)	20 (7.87%)	132 (8.53%)	27 (8.08%)	12 (6.03%)
Uyghur	7683 (92.02%)	470 (92.34%)	62 (91.18%)	231 (90.94%)	1414 (91.34%)	304 (91.02%)	187 (93.97%)
Other	5 (0.06%)	1 (0.02%)	0	3 (1.18%)	2 (0.13)	3 (0.90%)	0

### Diagnostic condition distribution

**Fig. 1:**
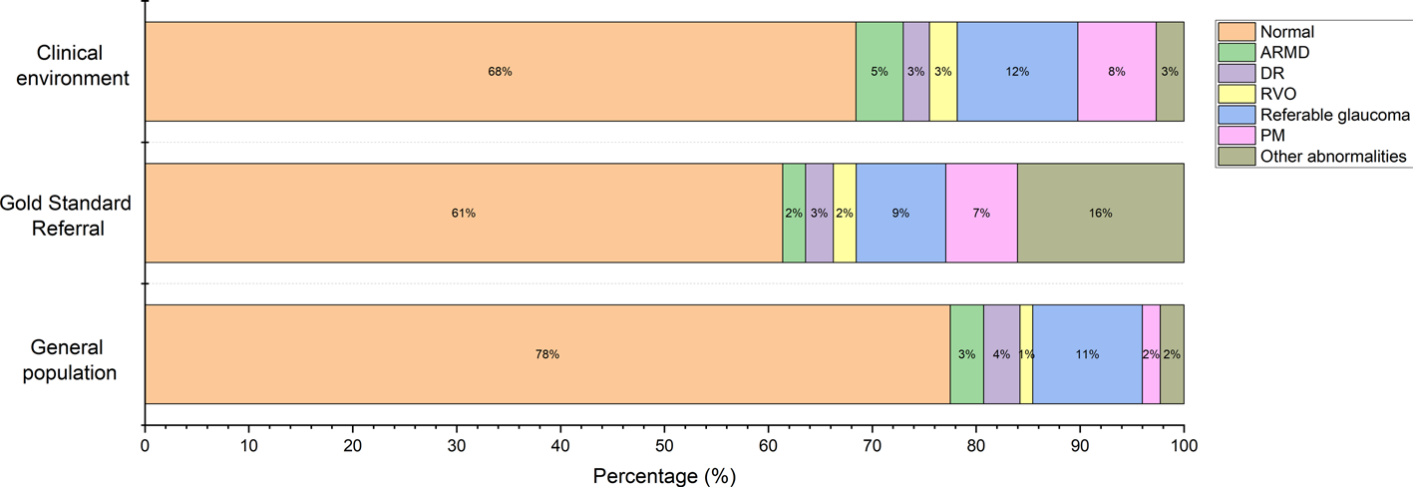
Percentage of diagnostic results for AI and gold standard referral. The percentages of 7 conditions in clinical application environment and population screening are shown and compared. *ARMD* age-related macular degeneration, *DR* diabetic retinopathy, *RVO* retinal vein occlusion, *PM* pathological myopia

**Fig. 2:**
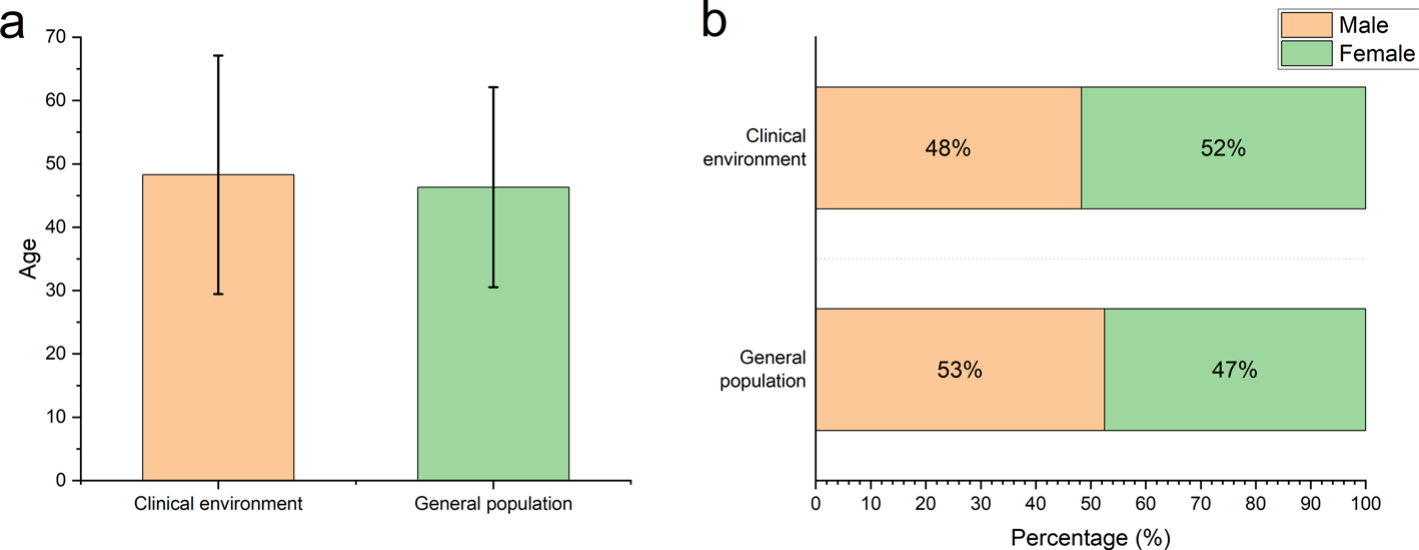
Comparison of age and sex in the clinical AI application environment and population screening. **a** Comparison of the average age in the clinical environment and population screening. **b** Comparison of the sex percentage in the clinical environment and population screening

The diagnostic results of AI included 436 normal, 29 ARMD, 16 DR, 17 RVO, 74 referable glaucoma, 48 PM and 17 other abnormalities, while the gold standard diagnostic results showed 391 normal, 14 ARMD, 17 DR, 14 RVO, 55 referable glaucoma, 44 PM and 102 other abnormalities. The diagnostic results of AI population screening included 15,779 normal, 653 ARMD, 713 DR, 248 RVO, 2146 referable glaucoma, 350 PM and 466 other abnormalities. The percentages of different diagnostic conditions in the two application environments were compared (Fig. 1). The comparisons for age and sex in the clinical AI evaluation and population screening was also analyzed (Fig. 2).

### Evaluation of AI application in the clinical environment

**Fig. 3:**
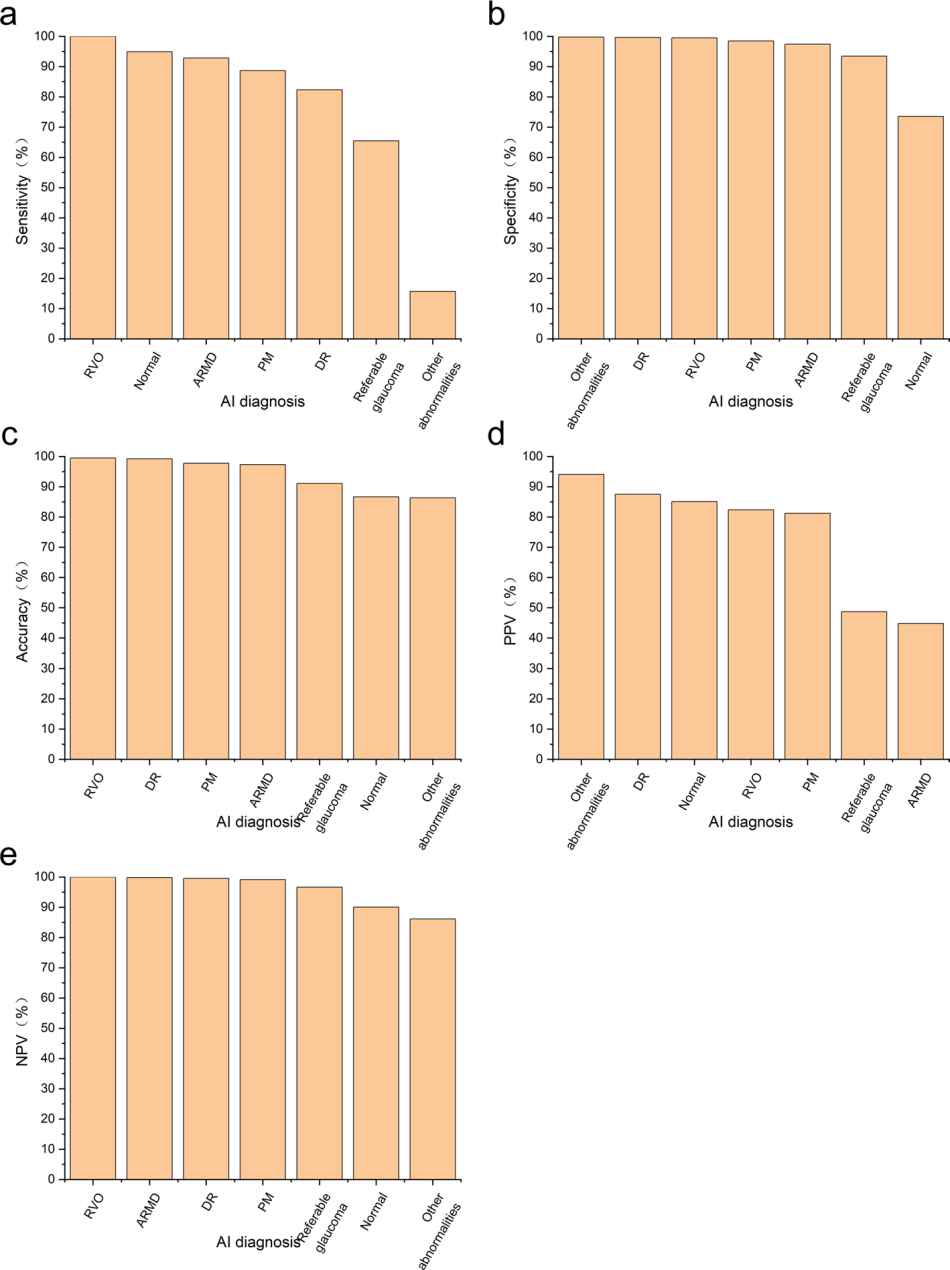
Comparison of different diagnostic results in AI clinical examination. **a** Sensitivity comparison of 7 diagnostic results. **b** Specificity comparison of 7 diagnostic results. **c** Accuracy comparison of 7 diagnostic results. **d** PPV comparison of 7 diagnostic results. **e** NPV comparison of 7 diagnostic results. PPV, positive predictive value; NPV, negative predictive value

**Fig. 4:**
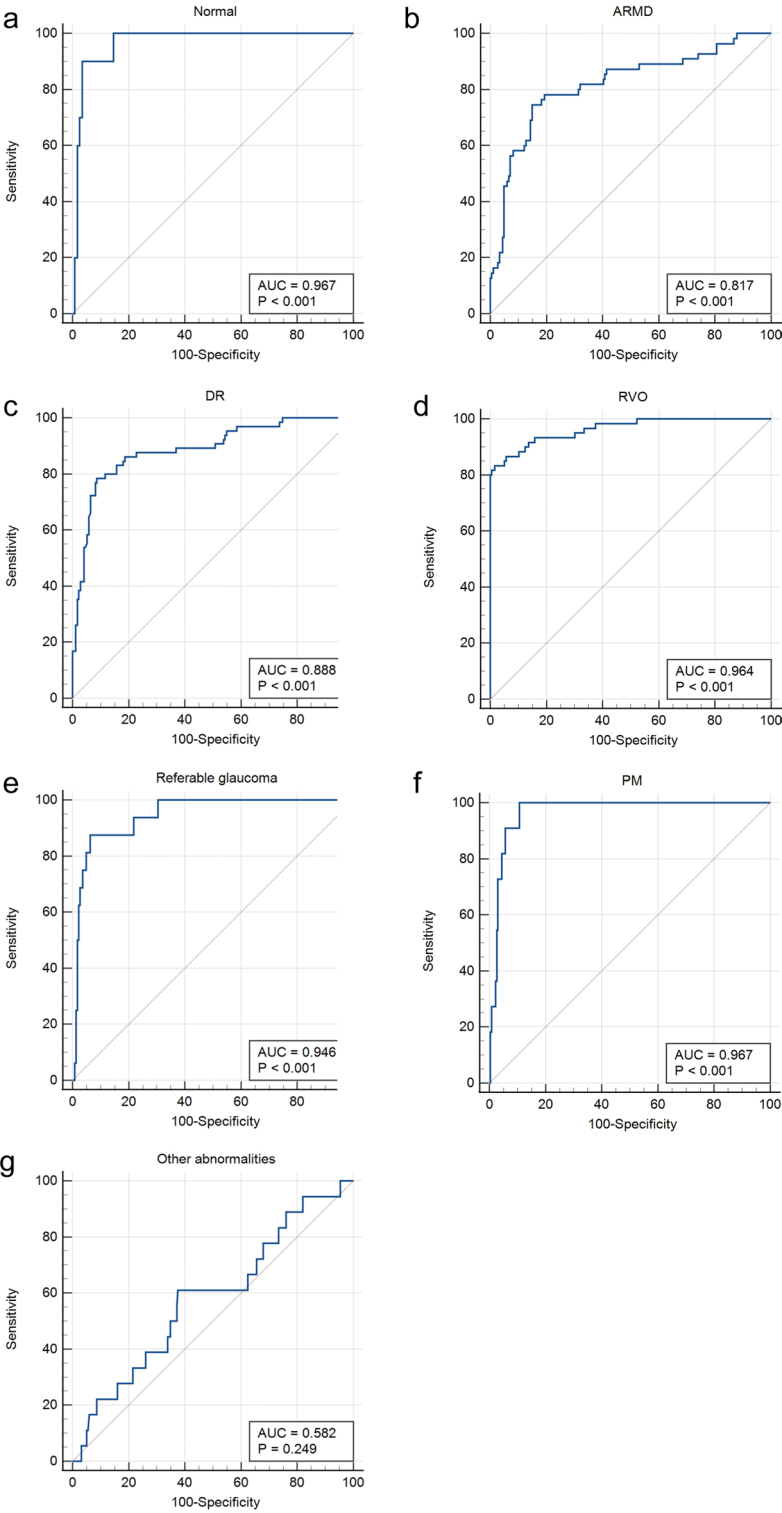
The receiver operating characteristic curve for every condition. **a** ROC curve and AUC for normal. **b** ROC curve and AUC for ARMD. **c** ROC curve and AUC for DR. **d** ROC curve and AUC for RVO. **e** ROC curve and AUC for referable glaucoma. **f** ROC curve and AUC for PM. **g** ROC curve and AUC for other abnormalities. AUC, areas under receiver operating characteristic curve

Regarding the validity of the AI, RVO showed the highest sensitivity and the lowest false negative rate (FNR) and negative likelihood ratio (LR-), while other abnormalities showed the highest specificity and the lowest false positive rate (FPR). DR had the highest positive likelihood ratio (LR +) (Table 2, Fig. 3). Regarding precision, RVO showed the highest accuracy and the strongest Kappa value. About application effect, RVO had the highest NPV, while other abnormalities had the highest PPV (Table 3, Fig. 3). For normal, ARMD, DR, RVO, Referable glaucoma and PM, the AI screening system achieved an area under the ROC curve greater than 0.8 (Fig. 4).

**Table 2:** The validity of AI for clinical examination

	Sensitivity (%)	FNR (%)	Specificity (%)	FPR (%)	LR +	LR−	Youden index
Normal	94.88	5.12	73.58	26.42	3.59	0.07	0.68
ARMD	92.86	7.14	97.43	2.57	36.13	0.07	0.90
DR	82.35	17.65	99.68	0.32	257.34	0.18	0.82
RVO	100.00	0.00	99.52	4.82	20.75	0.00	1.00
Referable glaucoma	65.45	34.55	93.47	6.53	10.02	0.37	0.59
PM	88.64	11.36	98.48	1.52	58.31	0.12	0.87
Other abnormalities	15.69	84.31	99.81	0.19	82.58	0.85	0.16
All fundus abnormalities	73.58	26.42	94.88	5.12	14.37	0.28	0.68

*ARMD* age-related macular degeneration, *DR* diabetic retinopathy, *RVO* retinal vein occlusion, *PM* pathological myopia, *FNR* false negative rate, *FPR* false positive rate, *LR + * positive likelihood ratio, *LR−* negative likelihood ratio

**Table 3:** Evaluation of the precision and application effect of AI

	Accuracy(%)	Kappa value	Conformance strength	PPV (%)	NPV (%)
Normal	86.66	0.709*	High	85.09	90.05
ARMD	97.33	0.593*	Middle	44.83	99.84
DR	99.21	0.844*	Strongest	87.50	99.52
RVO	99.53	0.901*	Strongest	82.35	100.00
Referable glaucoma	91.05	0.510*	Middle	48.65	96.63
PM	97.80	0.836*	Strongest	81.25	99.15
Other abnormalities	86.34	0.234*	Reasonable	94.12	86.13
Any fundus abnormalities	86.66	0.709*	High	90.05	85.09

*PPV* positive predictive value, *NPV* negative predictive value

*P < 0.001

## Discussion

It was previously demonstrated that, after training with images from common fundus imaging examinations such as CFP and OCT, an AI-based fundus diagnostic system could diagnose ocular fundus disorders like ARMD, DR [12] and glaucomatous optic neuropathy (GON). The substantial application value of AI-based screening systems for the early diagnosis of ocular fundus disorders has gained increasing attention [13]. There are many ways to examine the ocular fundus, and a previous study showed that an AI system based on OCT has sensitivity and specificity for high myopia that can exceed 80% [5]. However, to make a diagnosis, several image slices of a specified region needed to be scanned, which is unsuitable for fundus disease screening in primary medical institutions due to the low efficiency and equipment burden of the process. Additionally, multimodal imaging [14] is thorough and shows excellent performance for diagnosing ocular fundus abnormalities, but the combination of examination methods varies and depends on the specific diseases [15], making its application at primary hospitals more challenging. CFP, with the advantages of fewer images that need to be analyzed, ease of handling and low cost [16], has become the required test for ocular fundus examination and is more suitable for fundus screening in primary medical institutions [17].

There is currently no generally recognized standard for the validity of AI diagnosis systems. However, for AI-based DR diagnostic systems, screening guidelines suggest that sensitivity and specificity should be more than 80% [18]. Our study was a real-world study [19], and the AI-based fundus screening system we used in our study revealed that the sensitivity and specificity for ARMD, DR, RVO and PM were all above 80%, demonstrating the high degree of coincidence between AI and the gold standard referral diagnosis. The accuracies of diagnosing ARMD, DR, RVO and PM also surpassed 80%, showing the high stability of repeat examination for the same color fundus image. DR, RVO and PM all showed PPV and NPV values that exceeded 80%. After examining indicators for validity, precision and application effect in the clinical application environment, it was determined that among the five disorders, the screening capabilities of the AI system for DR, RVO and PM were better. Currently, only one CFP-based screening system for DR has been authorized by Food and Drug Administration (FDA), with the sensitivity and specificity surpassing 80%, which proved the feasibility of screening for DR in primary care settings [1]. In addition, the sensitivity and specificity of the AI system based on CFP for RVO [17], PM [20] and ARMD [2] was also able to exceed 80%, which was in line with our results.

Previously, an AI system based on CFP for multiple ocular fundus diseases only had an accuracy of 30.5% and Kappa value of 0.224 [21]. Additionally, there is an AI diagnostic system that can recognize a variety of fundus abnormalities, including hemorrhage, drusen, and any vascular abnormality [22]. For clinicians who would become fatigued after long working hours, this system was helpful for finding every subtle abnormality. However, due to the Al’s inability to provide a definite illness classification and the fact that the same fundus abnormality could be present in a variety of ocular fundus diseases, its application value in primary care was constrained. When 6 abnormal categories were included, the accuracy of the AI system in our study was up to 86.66% and the Kappa value was 0.709. This reflected the higher conformity, precision and more stable diagnosis for our AI system. Our results showed that the percentages of different conditions were similar in the clinical AI application environment and population screening for ARMD, DR, RVO and referable glaucoma. The percentages of PM and other abnormalities in population screening were only slightly less than those in the ophthalmic clinic. These results all demonstrated the beneficial effect of our AI-based fundus system for fundus screening and epidemiological research. Because there were more healthy people in the physical examination center than in the ophthalmic clinic, the percentage of normal in the population screening was higher than that in the clinical environment.

The specificity, accuracy and middle Kappa conformity strength of the AI system in clinical environment evaluation for referable glaucoma all exceeded 80%; however, the sensitivity was only 65.45%. Regarding the indicators of the application effect, the NPVs of ARMD and referable glaucoma were greater than 80%, while the PPVs of two abnormalities were low, with a PPV of 48.65% for referable glaucoma and 44.83% for ARMD. The sensitivity of referable glaucoma was low, and the FNR was 34.55%. The reasons are analyzed as below. The acute angle-closure glaucoma patients in the preclinical phase who did not have ocular fundus abnormalities and the eyes that had undergone anti-glaucoma surgery and had a relatively normal optic nerve made up the majority of the missed referable glaucoma cases. Additionally, early GON has a similar appearance to a normal optic disc, and ocular fundus injury in glaucoma patients with a small optic disc is difficult to detect, making it challenging to diagnose. There is a sequence for the optic disc structure injury and vision field defects, and the fluctuation often occurs in the early stage of vision field defects. Therefore, missed diagnosis occurs when vision field defects are present, but the optic nerve head (ONH) injury is not obvious. The low PPV of referable glaucoma was mainly due to optic atrophy, normal eyes and PM, which was similar to the results of Li et al. [6]. In addition to GON, other conditions, such as various optic neuropathies, trauma and hereditary diseases, could also contribute to optic atrophy. The form of the optic disc clearly varies in normal eyes, and physiologic large cup is often misdiagnosed as glaucoma. All of these factors can lead to misdiagnosis in the real clinical environment. When the pale area of the optic disc is larger than the sink region, diseases other than glaucoma should be considered. Moreover, PM can be misdiagnosed as glaucoma because of lateral optic atrophy, and myopia is one of the risk factors for open-angle glaucoma [23].

The PPV of ARMD in AI clinical examination is low, and misdiagnoses mainly included retinal detachment (RD), macular hole (MH) and macular epiretinal membrane. The fact that ARMD is one of the age-related degenerative pathological changes that can result in MH [24] is a significant factor contributing to misdiagnosis. In addition, ARMD can be classified as dry (atrophy type) and wet (exudative type) depending on the presence of hemorrhage, exudation and edema. Dry ARMD can display “gold foil”-like light reflection that is similar to the macular epiretinal membrane, while wet ARMD can exhibit serous and/or hemorrhagic RD. Low PPV may be caused by both the diversity of ARMD fundus abnormalities and the limitations of two-dimensional color fundus images. For other abnormalities, the specificity, accuracy, PPV and NPV were more than 80%, while the sensitivity was only 15.69%, and the Kappa value was reasonable. All of these results demonstrated that the AI-based fundus screening system had a relatively limited capability of recognizing ocular fundus abnormalities other than ARMD, DR, RVO, referable glaucoma and PM. The missed diagnosis of other abnormalities for the AI system mainly occurred in optic atrophy cases, which could be caused by a number of diseases, such as diabetes. There were 45 patients with optic atrophy who were under 50 years old, had no systemic or ocular medical history, and required further examination to determine the cause of their optic atrophy. For normal subjects, the sensitivity, accuracy, PPV and NPV exceeded 80%, and the Kappa conformation strength was high. However, the specificity was relatively low, 73.58%, demonstrating that AI system misdiagnosed some abnormal eyes, which mainly included diagnoses of glaucoma with inconspicuous GON and optic atrophy that should been classified as other abnormalities.

There was a preexisting AI system based on CFP designed to identify 4 diseases from 420 retinal images, such as DR and RVO [25]. The sensitivity, specificity and accuracy were all above 80%, demonstrating the broad application potential of this AI-based fundus diagnostic system. In this study, we proved the better application value of our proposed AI-based fundus screening system by choosing a larger sample size, more plentiful disease types, and collecting data from both a real clinical environment, and conducting a population screening of a large sample. Our study still has some limitations. Firstly, this study’s glaucoma screening effectiveness was insufficient. Our algorithm for learning cup–disc ratio is currently being updated. In addition to CFP, we take into account algorithm learning to combine anterior segment ocular manifestations and the vision field [26]. Second, our system needs to output more accurate and comprehensive diagnoses. We must provide more precise disorder stages, such as those for DR [27]. Every disorder that can be diagnosed from a color fundus image must be included in our future AI screening system. It has been proven that CFP can be adopted in AI-based cataract diagnostic systems [28], and sometimes multiple diseases can appear in a color fundus image simultaneously, such as RVO combined with glaucoma. Finally, considering the intense relationship between ocular diseases and hereditary or systematic diseases, we should combine our algorithm with systemic indicators such as glycosylated hemoglobin (HbA1c) [29] when diagnosing DR. Additionally, we also need to diagnose any possible systematic diseases from ophthalmic images [30].

## Conclusions

The AI-based fundus screening system used in this study is able to distinguish between normal and abnormal ocular fundus as well as diagnose 5 prevalent ocular conditions. Combining the indicators of validity, precision and application effect in a real clinical environment, the screening efficacies for DR, RVO and PM are more favorable. The system’s ability to recognize ARMD and referable glaucoma still need to be improved. The epidemiologic data from the ophthalmic clinic reveal that certain patients with optic atrophy are young and lack ophthalmic or systemic disorders, which likely indicates an epidemiologic trait in the local area and requires further study to determine the cause.

## Methods

### Image collection

**Fig. 5:**
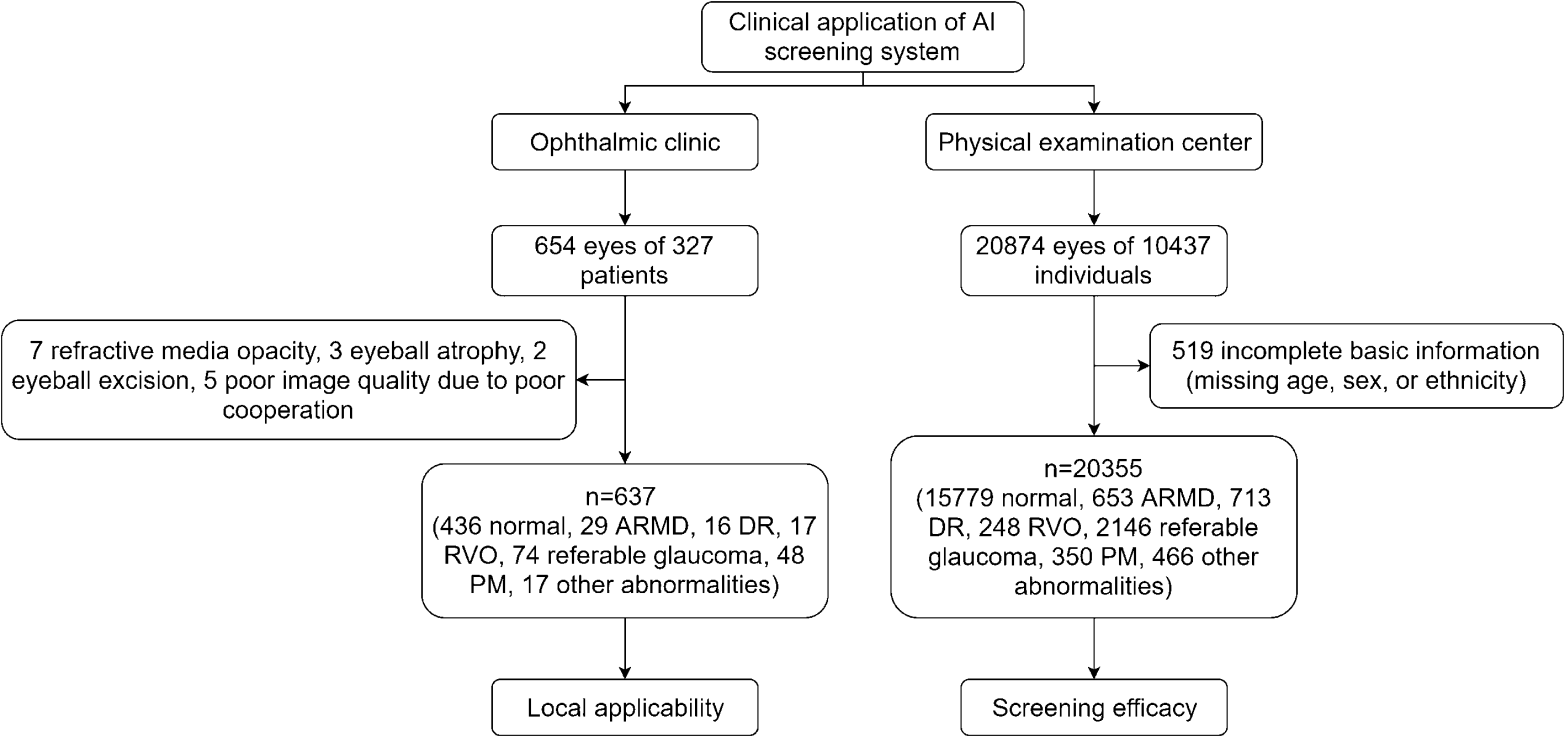
Flowchart for testing the AI fundus screening system

All investigations were carried out according to the Declaration of Helsinki and were approved by the institutional review board of the First People’s Hospital of Kashi Prefecture (No. 2019ksd-86). Every participant provided informed consent. To evaluate the clinical application of AI, 637 color fundus images of 327 patients from an ophthalmic clinic were collected. A total of 20,355 color fundus images of 10,437 individuals from a physical examination center were included for the evaluation of the general population screening. Only one image was included for each examined eye. Eyes with one of the following criteria were excluded: refractive medium opacity, eyeball excision, atrophy of the eyeball, and poor cooperation leading to inferior image quality. The flowchart in Fig. 5 depicts the inclusion procedure.

### AI system workflow

Color fundus images were captured with a fully automatic nonmydriatic fundus camera (RetiCam 3100, Shenzhen New Vision Technology Co., Ltd.) with installed AI-based fundus screening software (Kangrui Intelligent Technology Co., Ltd., Guangzhou, China). The AI system’s standard color fundus image met the following criteria [31]: (1) the photo was taken using a single field of view with at least a 45° retinal scope. (2) The center of the shooting field was defined as the midpoint of the connecting line between the fovea and the optic disc. (3) The focus of the image was between the fovea and the optic disc. (4) The structure of the optic disc, the fovea and the first superior and inferior branches of the retinal vascular arch could be observed in the image. (5) The image showed the 2-diameter region surrounding the optic disc. (6) The image had a 1024 × 1024 resolution.

When the eligible color fundus images were input into the AI software, the system could output one of the following conditions: normal, ARMD, DR, RVO, referable glaucoma, PM and other abnormalities. The gold standard referral for every condition was evaluated by two highly competent ophthalmic chief clinicians with more than 20 years of clinical experience according to the international diagnostic criteria (Table 4). When the two medical professionals disagreed, the gold standard referral outcome was determined by a third ophthalmic chief clinician with more than 20 years of clinical experience according to international guidelines.

**Table 4:** Diagnostic criteria for different conditions

Condition	Diagnostic criteria
Normal	Eyes with healthy ocular fundus, which includes normal fovea and optic disc, as well as normal retinal and choroidal vessels [32]
ARMD	A degenerative condition that affects older people and causes drusen and choroidal neovascularization in the macula, which are symptoms that can lead to progressive vision loss [33]. The diagnostic methods include CFP, fluorescein angiogenesis (FA), indocyanine green angiography (ICGA), optical coherence tomography (OCT) and optical coherence tomography angiography (OCTA)
DR	Patients with diabetes who have ocular fundus abnormalities such as microaneurysms, intraretinal hemorrhages and neovascularization [34]. Patients do not experience subjective symptoms in the early stages, and after developing maculopathy, they experience variable degrees of vision loss
RVO	The obstruction of retinal vein, which can result in retinal ischemia, retinal exudates, macular edema and/or intraretinal hemorrhages, and then cause vision loss and visual field defect [35]
Referable glaucoma	Glaucomatous diagnosis are made based on both anatomical and functional evidence, mainly including the glaucomatous field defects and optic cup enlargement [36] (Because glaucoma cannot be diagnosed solely by CFP and requires clinical diagnosis by subjective and objective testing, the diagnostic condition “referable glaucoma” of AI screening system was created. In addition, the glaucoma diagnosis criterion served as the benchmark for evaluating AI system.)
PM	Pathological myopia refers to the presence of ocular fundus complications, including posterior staphyloma, myopic maculopathy that is equal to or more serious than diffuse choroidal atrophy, and associated optic neuropathy [23]
Other abnormalities	Other ocular fundus abnormalities except the above 5 conditions (ARMD, DR, RVO, referable glaucoma and PM)

### Statistical analysis

All statistical analyses were performed using SPSS Statistics version 26 (IBM Corporation). For clinical application examination, evaluation indicators include validity, precision and application effect. The validity and degree of coincidence between the AI and human diagnoses were measured with sensitivity, false negative rate (FNR), specificity, false positive rate (FPR), positive likelihood ratio (LR +), negative likelihood ratio (LR-), Youden index and area under receiver operating characteristic (ROC) curve (AUC). The precision was evaluated by accuracy and Kappa value. The conformance strength of Kappa was determined in reference to the Landis & Koch criterion (Table 5). The application effect was evaluated by the positive predictive value (PPV) and negative predictive value (NPV). The figures were created in Origin 2022b (OriginLab Corporation). All the hypothesis tests were 2-sided, and a *P* value of less than 0.05 was considered significant.

**Table 5:** Evaluation criterion for Kappa value

Kappa value	Conformance strength	Kappa value	Conformance strength
< 0.00	Weak	0.41 ~ 0.60	Middle
0.00–0.20	Mild	0.61 ~ 0.80	High
0.21–0.41	Reasonable	0.81 ~ 1.00	Strongest

## Data Availability

The datasets of clinical examination and population screening that we used in this study are sourced from the First People’s Hospital of Kashi Prefecture. The availability of datasets is restricted under the agreement for the current study, so we are unable to make the datasets publicly available immediately. However, datasets and corresponding annotations can be made available upon reasonable request and with the permission of the regular and respectable research institutes. Please contact the first author Shujuan Cao (caoshujuancsj@126.com) for the request of data access.

## Abbreviations

AI: Artificial intelligence
AUC: Areas under receiver operating characteristic curve
ROC: Receiver operating characteristic
ARMD: Age-related macular degeneration
DR: Diabetic retinopathy
GON: Glaucomatous optic neuropathy
CFP: Color fundus photography
OCT: Optical coherence tomography
DL: Deep learning
RVO: Retinal vein obstruction
PM: Pathological myopia
FNR: False negative rate
FPR: False positive rate
LR +: Positive likelihood ratio
LR−: Negative likelihood ratio
PPV: Positive predictive value
NPV: Negative predictive value
FDA: Food and Drug Administration
ONH: Optic nerve head
RD: Retinal detachment
MH: Macular hole
HbA1c: Glycosylated hemoglobin
